# Targeting of the breast cancer microenvironment with a potent and linkable oxindole based antiangiogenic small molecule

**DOI:** 10.18632/oncotarget.16763

**Published:** 2017-03-31

**Authors:** Orestis Argyros, Theodoros Karampelas, Aimilia Varela, Xenophon Asvos, Athanasios Papakyriakou, Adamantia Agalou, Dimitris Beis, Constantinos H. Davos, Demosthenes Fokas, Constantin Tamvakopoulos

**Affiliations:** ^1^ Division of Pharmacology-Pharmacotechnology, Biomedical Research Foundation Academy of Athens, Athens, 11527, Greece; ^2^ Cardiovascular Research Laboratory, Clinical, Experimental Surgery and Translational Research Center, Biomedical Research Foundation Academy of Athens, Athens, 11527, Greece; ^3^ Laboratory of Medicinal Chemistry, Department of Materials Science and Engineering, University of Ioannina, Ioannina, 45110, Greece; ^4^ Laboratory of Chemical Biology of Natural Products and Designed Molecules, Institute of Physical Chemistry, N.C.S.R “Demokritos”, Athens, 15310, Greece; ^5^ Developmental Biology, Biomedical Research Foundation Academy of Athens, Athens, 11527, Greece

**Keywords:** angiogenesis, sunitinib analogue, tumor targeting, breast cancer, tumor microenvironment

## Abstract

The clinical efficacy of antiangiogenic small molecules (*e.g*., sunitinib) in breast carcinoma has largely failed with substantial off-target toxicity. We rationally designed and evaluated preclinically a novel sunitinib analogue, SAP, with favourable pharmacological properties and the ability to be readily conjugated to a targeting peptide or antibody for active tumour targeting.

SAP was evaluated *in silico* and *in vitro* in order to verify target engagement (*e.g*., VEGFR2). Pharmacokinetic and biodistribution parameters were determined in mice using LC-MS/MS. SAP efficacy was tested in two breast cancer xenograft and two syngeneic animal models and pharmacodynamic evaluation was accomplished using phosphokinase assays and immunohistochemistry. Cardiac and blood toxicity of SAP were also monitored.

SAP retained the antiangiogenic and cytotoxic properties of the parental molecule with an increased blood exposure and tumor accumulation compared to sunitinib. SAP proved efficacious in all animal models. Tumors from SAP treated animals had significantly decreased Ki-67 and CD31 markers and reduced levels of phosphorylated AKT, ERK and S6 compared to vehicle treated animals. In mice dosed with SAP there was negligible hematotoxicity, while cardiac function measurements showed a reduction in the percentage left ventricular fractional shortening compared to vehicle treated animals.

In conclusion, SAP is a novel rationally designed conjugatable small antiangiogenic molecule, efficacious in preclinical models of breast cancer.

## INTRODUCTION

Breast cancer (BrCa) is the most common malignancy among women and is clinically stratified into three major types depending on the expression of the estrogen (ER) and progesterone receptor (PR), or amplification/overexpression of the receptor tyrosine kinase (RTK) HER2/neu [1, 2]. In contrast, the triple-negative breast cancer (TNBC) type lacks expression of any of these three receptors and is generally characterized by poor prognosis [3].

A hallmark of BrCa is angiogenesis, a tightly orchestrated process between stromal and tumor epithelial cells [6]. Key stimuli are provided by proangiogenic factors such as the vascular endothelial (VEGF) and the platelet derived growth factor (PDGF) together with their respective receptors. Preclinical studies have shown that inhibition of the VEGF pathway impedes tumor growth and clinically the VEGF-neutralizing antibody bevacizumab was the first antiangiogenic treatment in BrCa due to the E2100 trial [7]. Subsequent failure to reproduce the results of the E2100 trial lead to withdrawal of the indication of bevacizumab in BrCa treatment [8].

Antiangiogenic small molecules (*e.g*., sunitinib) have initially produced a robust preclinical efficacy in BrCa [9–12] justifying the initiation of several clinical trials, where sunitinib was evaluated in combination therapies with cytotoxic molecules. Unfortunately, the majority of such combinational efforts failed to meet the designed primary end points and trials were discontinued, either due to the lack of efficacy, or dose limitations due to toxicity [13]. For example, the SUN1064 study of sunitinib in combination with docetaxel in BrCa patients did not show a statistically significant improvement in PFS compared with docetaxel alone [14]. A similar result was obtained in the SUN1099 study of sunitinib combined with capecitabine [15]. However, a sophisticated treatment scheme employing sunitinib co-administration with trastuzumab (an antibody targeting the HER2) generated encouraging results in a phase II study of HER2 positive patients [16]. In addition, the recent initiation of a clinical trial (NCT02074878) in TNBC patients where sunitinib was administered with crizotinib (a MET inhibitor) highlighted that sunitinib still holds promise in BrCa clinical practice and further efforts are necessary especially in hard to treat TNBC and HER2 positive patients. The details behind the clinical shortcomings of sunitinib in BrCa are not entirely clear but suggested explanations include the growing evidence that BrCa angiogenesis is driven by more than just VEGF, so inhibition of many other proangiogenic kinases might be necessary; the activation of cancer stem cells that overcome tumor remission [10]; and certainly the limitation of administering a higher drug dose due to off-target toxicities such as left ventricular (LV) dysfunction and overt heart failure frequently encountered due to depletion of coronary microvascular pericytes [17].

We have recently demonstrated that conjugation of a rationally designed analogue of sunitinib (called SAN1) to a decapeptide moiety (generating SAN1GSC) alleviated such toxicity possibly due to the ability of SAN1GSC to selectively deliver the pharmacophore to the tumor microenvironment, as shown in a castration resistant prostate cancer animal model [18]. SAN1 was also active in BrCa cell lines but its development encountered various hurdles, namely a low synthetic yield (71%), and the limited conjugation options that the free hydroxyl group of SAN1 allowed for. Thus we have now opted to generate a new sunitinib analogue, to pharmacologically equivalent to sunitinib or SAN1 that will also allow for additional conjugation possibilites. The pivotal therapeutic role of conjugated small molecules in BrCa has been strengthened by the recent success of T-DM1, allowing us to design a distinct therapeutic approach based on implementing an antiangiogenic agent conjugated to a targeting peptide or antibody in order to treat BrCa at the tumor microenvironment rather than solely at the individual cancer cell.

Herein we present the synthesis and preclinical evaluation of a novel piperazine based analogue of sunitinib (called SAP). SAP was rationally designed to maintain the potency of sunitinib but with the advantage of targeted delivery by allowing for swift amine-based conjugation chemistries with peptides or antibodies. In addition, the piperazine functional group of SAP is also predicted to augment the compound's anticancer effects [24–26], while pharmacologically SAP is characterised by lower cLogP, higher polar surface area and improved aqueous solubility.

## RESULTS

### Synthesis of SAP

**Figure 1:**
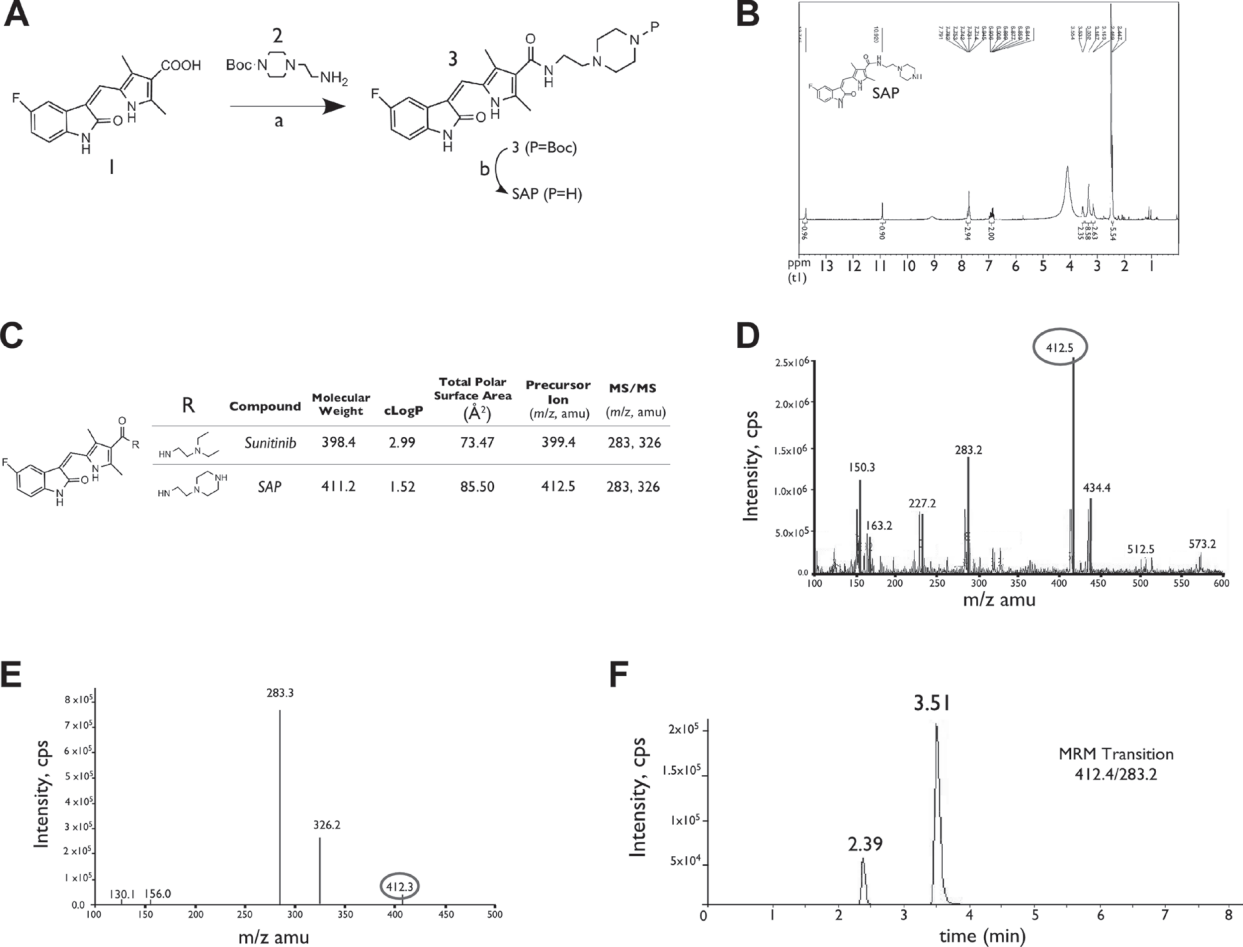
Synthesis and characterization of SAP (**A**) Synthesis of SAP from the available acid intermediate 1. Reagents and conditions: (a) DMF, EDCI, HOBt, NE_t3_, rt: room temperature; (b) CH_2_C_l2_, TFA, rt. (**B**) NMR spectrum of SAP. (**C**) Key physical properties for SAP and sunitinib. (**D**) Representative positive electrospray ionization mass spectra of SAP showing the main ionized form (M+1, *m/z* 412.5). (**E**) MS/MS analysis of the parent ion with *m/z* 412.5 and the formation of product ions with *m/z* 283.3 and *m/z* 326.2 (**F**) LC/MRM chromatogram of SAP, demonstrating the presence of E and Z geometric isomers.

The synthetic route for SAP is shown in Figure 1A while the accurate chemical structure was verified by ^1^H Nuclear Magnetic Resonance (NMR) (Figure 1B). SAP retained the indolin-2-one core of sunitinib responsible for RTK inhibition, while it provided a handle for conjugation to a targeting peptide or antibody through the amino group of piperazine. Figure 1C depicts the MS features as well as some key properties of SAP. The lipophilicity of SAP was calculated to be lower than sunitinib (cLog*P* = 1.5 vs 2.9 of sunitinib), and the calculated 2D PSA of SAP is higher in comparison to sunitinib (85.5 Å^2^ vs 73.5 Å^2^ of sunitinib). The calculated percent absorption (%ABS = 80, calculated using: %ABS = 109 – 0.345 PSA), and the degree of blood brain barrier permeability (logBB = –0.9 calculated using: logBB = –0.0148 PSA + 0.152 cLogP + 0.139) suggest a good absorption and poor brain permeability profile of SAP with respect to other angiogenesis inhibitors [27]. A representative mass spectrum for SAP and an LC-MS/MS chromatogram (depicting the Z and E isomers of SAP) are shown in Figure 1D–1F.

### Molecular docking

**Figure 2:**
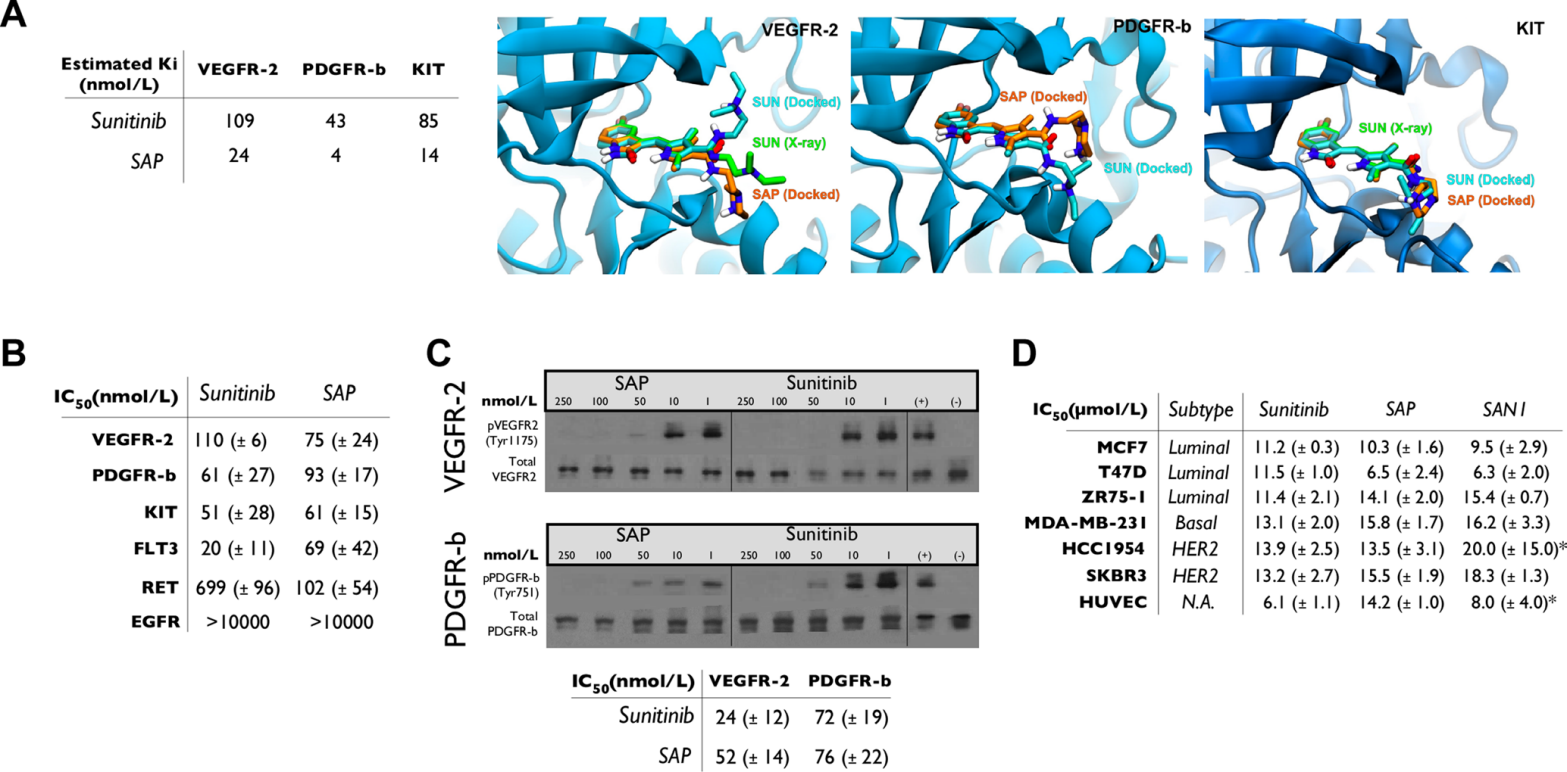
*In silico* and *in vitro* analysis of SAP (**A**) Docking results for SAP and sunitinib. Molecular models of the ATP-binding site of VEGFR-2, PDGFR-β and KIT illustrating the predicted bound poses of sunitinib (SUN, cyan sticks) and SAP (orange sticks) in comparison with the crystallographic conformation of sunitinib (SUN, green sticks) of VEGFR2 and KIT. (**B**) Summary of *in vitro* kinase activity in multiple RTKs in the presence of sunitinib or SAP (± SD). (**C**) Cell based autophosphorylation assay for VEGFR-2 and PDGFR-β in the presence of sunitinib or SAP (± SD). (**D**) MTT cytotoxicity assay in a panel of BrCa cell lines and HUVEC. *historical data. N.A: Not Active.

Molecular docking calculations were performed for SAP in comparison with sunitinib, which were based on the X-ray structures of VEGFR-2 and KIT complexes with sunitinib, and a homology model of PDGFR-β (Supporting Information SI2). The inhibition constants of SAP and sunitinib were estimated to be comparable (at the low nanomolar range), and representative molecular models of SAP bound to the ATP-binding site of SAP complexed with these RTKs are shown in Figure 2A.

### Biochemical kinase assay

The potency of SAP was evaluated in biochemical assays employing the known RTK targets of sunitinib (VEGFR-2, PDGFR-β, KIT, FLT3 and RET) and against the EGFR as a negative control (Figure 2B). SAP inhibited most of these RTKs with comparable affinity with respect to sunitinib (IC_50_ = 61–102 nmol/L), a result confirming out design strategy. An interesting observation was the superior potency of SAP over sunitinib in inhibiting RET (IC_50_ = 102 vs 699 nmol/L), a finding that merits further investigation.

### Cellular ligand-dependent phosphorylation assay

The inhibition of phosphorylation of VEGFR-2 and PDGFR-β was further confirmed in HUVEC and NIH/3T3 cells, using a Western blot based cellular ligand-dependent phosphorylation assay (Figure 2C). The calculated cellular IC_50_ values for sunitinib were 24 ± 12 nmol/L (for VEGFR-2) and 72 ± 19 nmol/L (for PDGFR-β) respectively. Similar potencies were observed for SAP with cellular IC_50_ values of 52 ± 14 nmol/L for VEGFR-2 and 76 ± 22 nmol/L for PDGFR-β.

### Cytotoxicity and antimetastatic cellular studies

The antiproliferative effect of SAP was assessed in several cell lines including three luminal (MCF7, T47D, ZR75-1), one basal TNBC (MDA-MB-231) and two HER2 amplified (HCC1954, SKBR3) BrCa subtypes, as well as the endothelial cell line HUVEC. Results and comparative IC_50_ values are presented in Figure 2D. SAP was equipotent to sunitinib, but also to our previously generated SAN1 molecule [18] in all cell lines with IC_50_ values ranging from 6.5 ± 2.4 μmol/L to 15.8 ± 1.7 μmol/L.

The antimetastatic potential of SAP was demonstrated in the MDA-MB-231 cell line using a wound healing assay and various drug concentrations. Results showed that SAP had similar potency to sunitinib at inhibiting cell migration based on cellular gap closure at 10 μmol/L (Supplementary Figure 1).

### Pharmacokinetic evaluation of SAP

**Figure 3:**
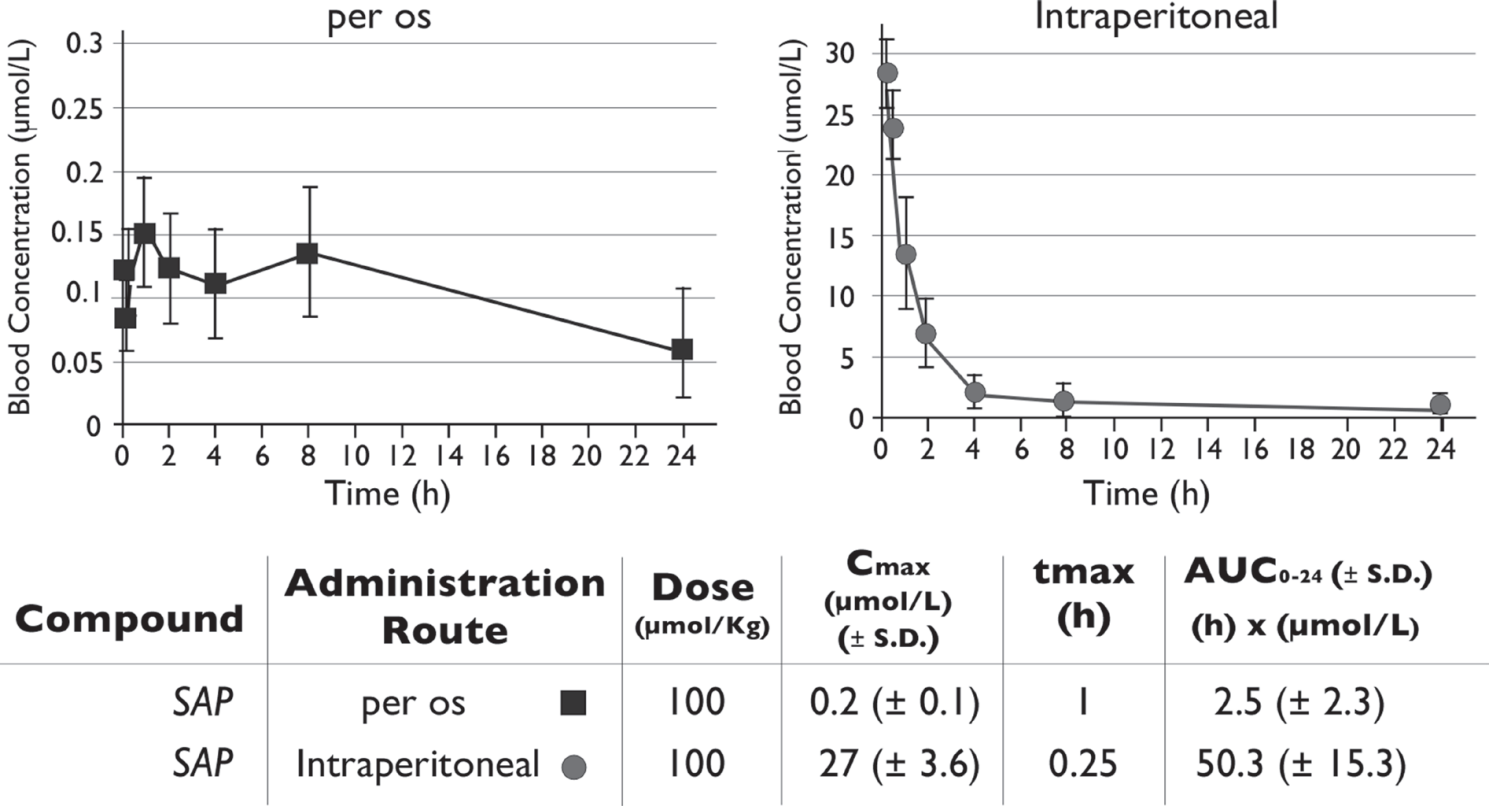
Pharmacokinetic evaluation of SAP Female NOD/SCID mice (*n* = 6) were dosed per os or IP with SAP (100 μmol/Kg) and blood samples were collected at selected time points. Drug levels of SAP were monitored by LC-MS/MS. The AUC for each treatment was calculated as a measure of drug exposure over time.

The *in vitro* efficacy of SAP was followed by its pharmacokinetic evaluation in mice. Initially, the PK parameters of SAP were compared in an oral versus IP administration (Figure 3). When dosed orally, SAP achieved maximum blood concentrations at 1h of 0.2 ± 0.1 μmol/L with an AUC_0−24 h_ of 2.5 ± 2.3 h × μmol/L. Higher blood exposure was observed following an IP administration, where SAP reached its highest blood concentrations at 0.25 h with a Cmax of 27 ± 3.6 μmol/L and an AUC_0–24 h_ of 50.3 ± 15.3 h × μmol/L. No signs of discomfort or overt toxicity were observed in any of the treated mice. Interestingly these pharmacokinetic measurements were superior to sunitinib when directly compared with previous results using the same administration route (IP) and an equimolar dose (Cmax for sunitinib was 6.5 ± 0.9 μmol/L at 0.25 h with an AUC_0−24 h_ of 21.4 ± 3.6 h x μmol/L) [18]. According to the biochemical assays SAP concentrations of approximately 0.1 μmol/L were needed for the inhibition of the target kinases (Figure 2A–2C). The pharmacokinetic experiments suggested that following IP dosing (at 100 umol/Kg), concentrations of at least 0.1 μmol/L could be sustained for more than 8h post-dose (SAP C_8 h_ = 0.93 ± 0.3 μmol/L), indicating that an IP dose at 100 umol/Kg should be the preferred route of administration for the subsequent preclinical efficacy studies in mice.

### *In vivo* antitumor efficacy in xenografted mice

**Figure 4:**
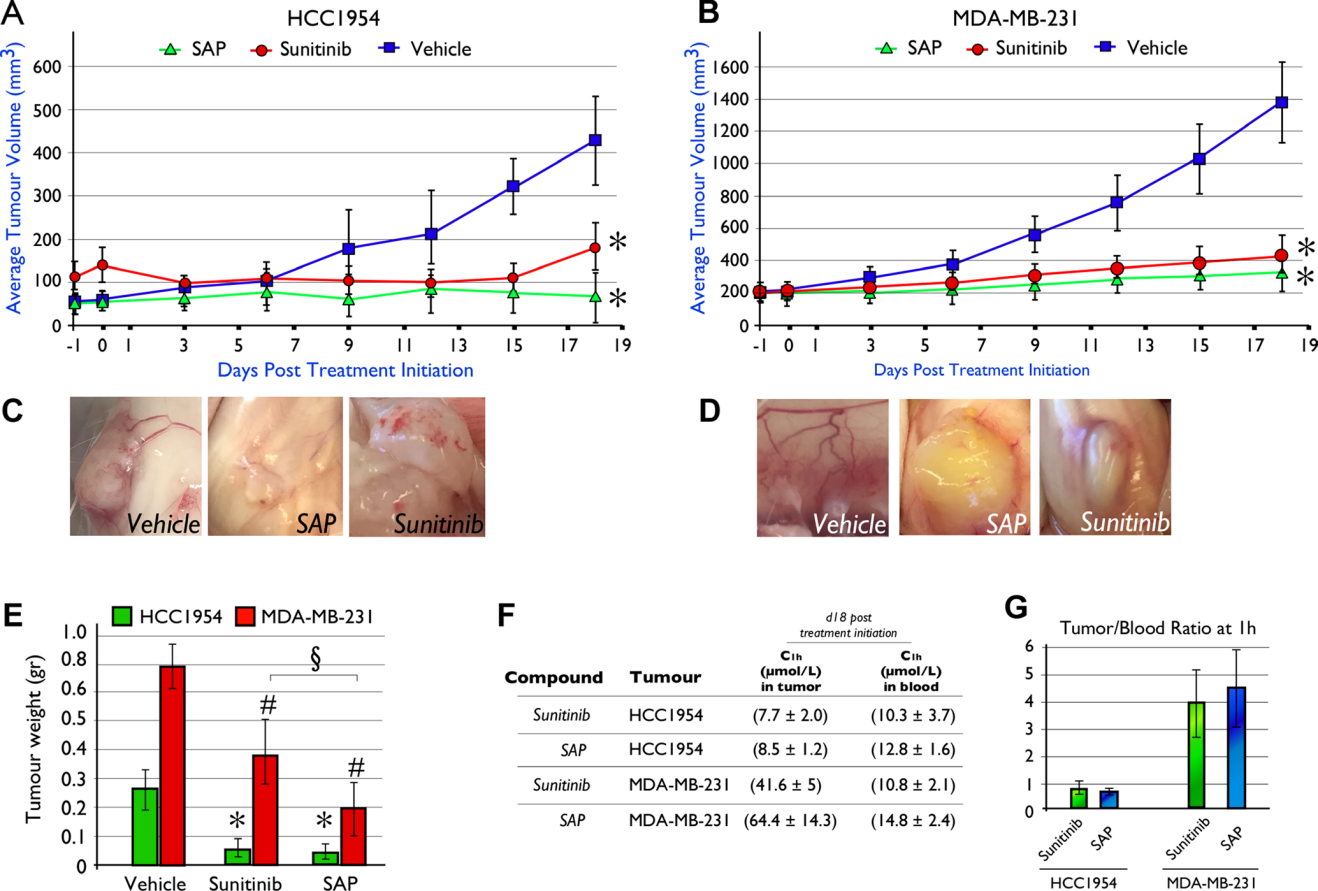
*In vivo* efficacy of SAP (100 μmol/Kg) versus equimolar amounts of sunitinib in NOD/SCID mice xenografted with (**A**) HCC1954 and (**B**) MDA-MB-231 cell lines. Mice were dosed (IP) daily with SAP, sunitinib or vehicle. Each point represents the mean of at least 10 tumor volumes resulting from at least five mice ± SD. * *P <* .001, compared with the vehicle group by using one way ANOVA followed by the post hoc Turkey-Kramer multiple comparison test (**C**) Evidence of reduced neo-angiogenesis in HCC1954 and (**D**) MDA-MB-231 xenografted tumors from SAP and sunitinib compared to vehicle treated mice. (**E**) Average tumor weight at day of sacrifice (d18) between treatment groups. Each bar is the average of 10 tumors for each treatment ± SD. The *for the HCC1954 and ^#^for the MDA-MB-231 cell line denote a *P <* .001, compared with their respective vehicle group while the § denotes a *P <* .001 between SAP and sunitinib, by using one way ANOVA followed by the post hoc Turkey-Kramer multiple comparison test. (**F**) Average intratumoral and blood drug levels as measured by LC-MS/MS at 1h post a final dose on d18 (± SD). (**G**) Tumor/blood (t/b) ratio for SAP and sunitinib from the LC-MS/MS measurements.

In order to determine whether SAP treatment could eradicate tumors as effectively as sunitinib in two aggressive (ER and PR independent) forms of BrCa, we generated xenografted mice bearing established tumors using the HCC1954 or MDA-MB-231 cell lines. Pharmacological treatment was initiated when tumors reached 100–150 mm^3^ for a total period of 18 days, using a dose of each molecule at 100 μmol/Kg/day. At the end of the treatment period (d18) for the HCC1954 animal model (Figure 4A) the average tumor size was 428 ± 101 mm^3^ for vehicle treated mice, significantly (*P <* .001) higher compared to SAP (76 ± 52 mm^3^) or sunitinib (152 ± 57 mm^3^) treated mice.

Similarly for MDA-MB-231 tumor bearing animals, both sunitinib and SAP were able to significantly (*P <* .001) delay tumor growth compared to vehicle treated animals. SAP treated animals had an average tumor size at d18 of 265 ± 98 mm^3^, sunitinib treated animals averaged 367 ± 117 mm^3^ while vehicle treated mice reached an average size of 1361 ± 250 mm^3^ (Figure 4B). Interestingly, a comparison between SAP and sunitinib at d18 revealed that SAP was statistically more efficacious (*P <* .001) Neo-angiogenesis was evident upon sacrifice in both BrCa xenograft models, for vehicle treated mice (Figure 4C–4D), while only traces of newly formed blood vessels were noted for SAP and sunitinib treated animals consistent with their antiangiogenic mechanism of action.

The average tumor weights upon sacrifice for the HCC1954 BrCa animal model were 0.26 ± 0.07 g for vehicle treated mice (Figure 4E), significantly (*P <* .001) heavier over SAP (0.05 ± 0.02 g) and sunitinib (0.07 ± 0.03 g). In the MDA-MB-231 animal model the respective tumor weights were 0.8 ± 0.2 g for vehicle, 0.2 ± 0.1 g for SAP and 0.4 ± 0.1 g for sunitinib, with both drugs at compared to vehicle treated animals (*P <* .001). In both animal models, no changes in animal total body weight (data not shown), or overt toxicity was observed.

Concentrations of sunitinib and SAP in blood and tumor tissue were measured at 1h after a final dose on d18 for both the HCC1954 and MDA-MB-231 animal models (Figure 4F). For the HCC1954 xenograft, the blood C_1 h_ was 10.3 (± 3.7) μmol/L for sunitinib and 12.8 (± 1.6) μmol/L for SAP, while the respective C_1 h_ in the tumor tissue was 7.7 (± 2.0) μmol/L for sunitinib and 8.5 (± 1.2) μM for SAP, generating a tumor/blood (t/b) ratio of 0.8 for sunitinib and 0.7 for SAP (Figure 4G). In the MDA-MB-231 animal model the C_1 h_ in the blood for sunitinib and SAP was 10.8 (± 2.1) μmol/L and 14.8 ( ± 2.4) μmol/L respectively, while the values for intratumoral drug levels were 41.6 (± 5) μmol/L and 64.4 (± 14.3) μmol/L. The t/b ratio for the MDA-MB-231 xenograft was 4.0 for sunitinib and 4.5 for SAP (Figure 4G). Despite the fact that sunitinib is more lipophilic in comparison to SAP (see clog *P* values), the high intratumoral concentrations of SAP in both xenografts may be due to a lower tissue clearance in comparison to sunitinib, a property that is favorable for tumor targeting.

### *In vivo* target modulation investigation

**Figure 5:**
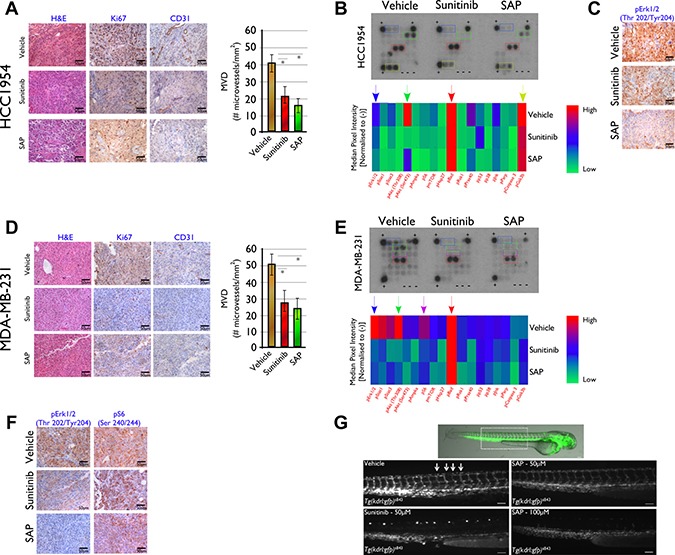
Histological and molecular investigation of SAP efficacy in (**A**–**C**) HCC1954 and (**D**–**F**) MDA-MB-231 tumor sections on d18 post treatment initiation. (A) Immunohistochemical analysis in HCC1954 tumor sections showed a marked decrease in cell proliferation (Ki-67) and angiogenesis (CD31) in SAP and sunitinib versus vehicle treated mice. (B) Heat map analysis of HCC1954 tissue lysate showing the *in vivo* phosphorylation status of 18 kinases for all treated mice. Data represent the average of 10 tumors from five mice for each treatment. Reduced levels of phosphorylated GSK3, Akt and Erk1/2 for SAP and sunitinib versus vehicle treated mice were detected and (C) verified for Erk1/2 by IHC. (D) A similar analysis in MDA-MB-231 xenografts revealed decreased proliferation and angiogenesis as well as (E) reduced phosphorylation of Akt, S6 and Erk1/2 for SAP and sunitinib over vehicle treated mice. (F) Reduced levels of phosphorylation for specific kinases were verified by IHC for Erk1/2 and S6. For each IHC photo, brown indicates DAB reaction product with representative x40 fields shown. Scale bar: 50 μm. (**G**) Inhibition of angiogenesis in Zebrafish from SAP and sunitinib. Overlay of fluorescent and bright field images of a vehicle treated (DMSO) *Tg(kdrl:gfp)*^s843^ embryo at 48hpf. The dotted line box indicates the location of the images shown in the other panels. Fluorescence microscopy images of 48 hpf embryos treated from 24 to 48 hpf with vehicle or 50 μM of SAP or sunitinib and 100 μM SAP. Vehicle and 50 μM SAP treated embryos exhibited uniform GFP expression in intersomitic vessels identified with white arrows. Sunitinib at 50 μM and SAP at 100 μM inhibited angiogenic sprouting in the zebrafish trunk. Scale bar 100 μm.

Insights into the molecular mechanism responsible for the *in vivo* efficacy of SAP and sunitinib were obtained by histological, immunohistochemical and target modulation analysis of tumors harvested from all animals on the day of sacrifice (d18) for both the HCC1954 and MDA-MB-231 animal models (Figure 5).

For HCC1954 treated mice, the IHC analysis in tumor sections using antibodies against Ki-67 and CD31 showed a marked reduction in cell proliferation and reduced angiogenesis in SAP and sunitinib compared to vehicle treated specimens (Figure 5A). The Ki-67 assessed PI of mice treated with SAP was 19.2 ± 4.9%, and 22.9 ± 7.8% for sunitinib (both at *P <* .001 over vehicle treated mice) whereas for vehicle treated mice it was 82.3 ± 11.1%. The average number of CD31+ cells in tumor sections of mice treated with SAP were 16.6 ± 7.1 (*P <* .001 versus vehicle treated mice), and 21.6 ± 9.0 (*P* < .001 compared to vehicle treated mice) for sunitinib versus 42.4 ± 8.3 for vehicle treated mice (Figure 5A).

In order to gain a deeper understanding of the mechanism of action of SAP in treated mice, we investigated the phosphorylation status of 18 kinases in extracts of tumor tissue and compared it to tumor extracts of vehicle and sunitinib treated mice (Figure 5B). A marked downregulation of pErk1/2 (Thr202/Tyr204), pAkt (Ser473) and pGSK3 (Ser9) was noted in SAP and sunitinib treated versus vehicle treated mice, consistent with the multikinase phosphorylation inhibition ability of sunitinib and the designed SAP analogue. High levels of pBad (Ser112) protein indicative of increased levels of apoptosis were seen in all treatment groups. The reduced levels of pErk1/2 (Thr202/Tyr204) were verified in subsequent IHC experiments (Figure 5C). A similar analysis was performed in tumor sections from the MDA-MB-231 xenografted mice (Figure 5D). In this case, the Ki-67 assessed PI of mice treated with SAP was 20.11 ± 4.5%, for sunitinib the respected value was 20.9 ± 5.6% (*P <* .001 for both drugs compared to vehicle treated mice), whereas for vehicle it was 97.4 ± 7.2%. The average number of CD31+ cells in tumor sections of mice treated with SAP were 25.7 ± 11.8, for sunitinib 28.1 ± 11.3 (both at *P <* .001 over vehicle treated mice) and for vehicle 50.6 ± 10.2.

Analysis of the phosphorylation status of 18 kinases in extracts of tumor tissue (Figure 5E), revealed a marked decrease mainly of pErk1/2 (Thr202/Tyr204), pAkt (Ser473) and pS6 (Ser235/236) in SAP and sunitinib treated versus vehicle treated mice. Again, high levels of pBad (Ser112) protein indicative of increased levels of apoptosis were seen in all treatment groups. The reduced levels of pErk1/2 (Thr202/Tyr204) and pS6 (Ser235/236) were verified in subsequent IHC experiments (Figure 5F).

As an additional *in vivo* model of SAP efficacy we employed the well established zebrafish angiogenesis inhibition assay. We used *Tg(kdrl:gfp)*^s843^ transgenic embryos treated from 24 to 48 hpf with equimolar amounts of either SAP or sunitinib. In *Tg(kdrl:gfp)*^s843^ transgenic embryos EGFP expression is driven by the kdrl endothelial specific promoter representative of the VEGF signaling and marks all endothelial cells. In zebrafish embryos the intersomitic vessels (ISVs) are first marked by *kdrl:gfp* labeling at 23 hpf, they are partially patent at 1.5 dpf and they show robust circulation by 2 dpf. The results of the effect of equimolar SAP and sunitinib are shown in Figure 5G. No toxic or off-target morphological phenotypes were observed after 24h treatment with 50 μM SAP or sunitinib. Control (DMSO treated) embryos had their ISVs arranged in an extremely regular array, while treatment with 50 μM sunitinib robustly inhibited angiogenic sprouting and no ISVs were present at 48 hpf. On the other hand, the ISVs of SAP treated embryos appeared to develop almost normally with minor ISV formation inhibition, which was more profound at 100 μM. The observed lower efficacy from SAP was clarified following whole embryo LC-MS/MS biodistribution measurements that demonstrated an approximate 50% uptake of the more hydrophilic SAP from fish embryos at 24h post drug exposure, in contrast to more than 90% uptake of the more lipophilic sunitinib (data not shown).

### Toxicity assessment in rodents

**Figure 6:**
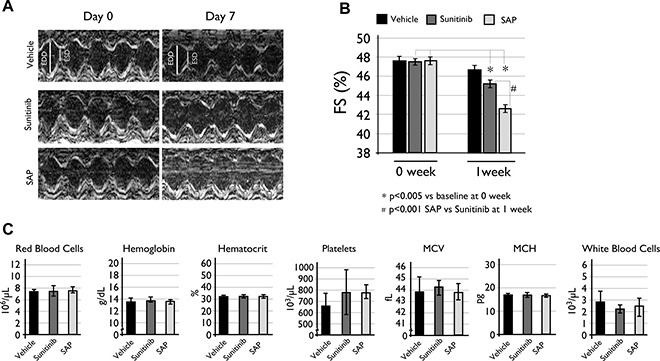
Toxicity evaluation of SAP (**A**) Cardiac LV function echocardiography measurements with representative M-mode echocardiograms and (**B**) %FS reduction compared to baseline levels at one week of treatment. Data are expressed as mean ± SD and a *P <* .005 value was considered statistically significant. (**C**) Hematological analysis of the same C57BL/6 mice used for the cardiotoxicity experiments. Each column is the mean of at least eight mice ± SD with a *P <* .05 value considered statistically significant. MCV: mean cell volume; MCH: mean cell hemoglobin.

Considering the known cardiotoxicity of sunitinib and related molecules [17, 28], we assessed the cardiac function of vehicle or compound treated C57BL/6 female mice. Results showed that both SAP and sunitinib had a statistically significant %FS (Fractional Shortening) reduction following treatment, with SAP appearing slightly more cardiotoxic than sunitinib (Figure 6A–6B). Mice treated with SAP had a %FS reduction from the baseline measurement of 47.48 ± 0.91 to 42.38 ± 0.76, *P <* .0001 at one week post treatment. The respective %FS value for sunitinib dropped from 47.48 ± 1.05 to 45.42 ± 0.11, *P* = .0047), whereas vehicle treated mice had a non-significant fall in %FS (from 47.49 ± 1.16 to 46.34 ± 0.56) as shown in Figure 6B.

Hematotoxicity of SAP and sunitinib was also evaluated for all treated mice at the end of the cardiotoxicity experiments. Results indicated a mild but statistically non-significant difference in white blood cell populations between vehicle (2.90 ± 0.8 × 10^3^ cells), sunitinib (2.24 ± 0.5 × 10^3^ cells) and SAP (2.54 ± 1.0 × 10^3^ cells) treated mice (Figure 6C). All other blood parameters measured remained constant, concluding that SAP had a similar hematotoxic profile with sunitinib under the specific experimental conditions.

### *In vivo* antitumor efficacy in syngeneic BrCa animal models

**Figure 7:**
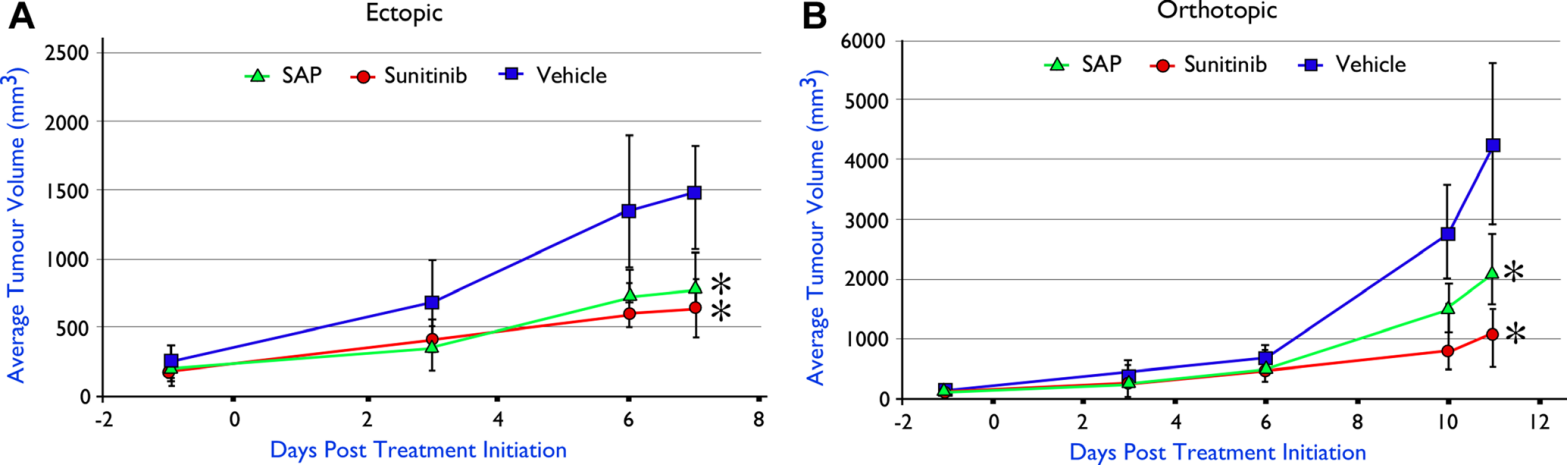
*In vivo* efficacy of SAP (100 μmol/Kg) versus equimolar amounts of sunitinib in C57BL/6 mice with implanted E0771 murine BrCa cell line (**A**) ectopically (*n* = 5) and (**B**) orthotopically (*n* = 6). Each point represents the mean of 10 (ectopic) and 6 (orthotopic) tumor volumes ± SD. * *P <* .001, compared with the vehicle group by using one way ANOVA followed by the post hoc Turkey-Kramer multiple comparison test.

Despite the efficacy and favourable safety profile of SAP in xenografted models of primary BrCa, a critical question that remained was its evaluation in a more clinically relevant disease model of BrCa that would involve interaction of cancer with the immune system, which is crucial for the progression of the disease. Thus we generated syngeneic animal models of BrCa in C57BL/6 immunocompetent mice using the E0771 BrCa murine cell line, which has been successfully used previously to evaluate sunitinib [29]. Equimolar amounts of SAP and sunitinib were evaluated in either the ectopic (Figure 7A) or orthotopic (Figure 7B) E0771 implantation setting using animals with identical genetic backgrounds, with results showing a significant tumour growth inhibition from both SAP and sunitinib (*P <* .01) in both cases in contrast to vehicle treated animals that had to be ethically sacrificed (at day 11) due to a heavy tumour burden. Upon sacrifice no macroscopic metastatic lesion was observed in any of the treated mice, despite the high metastatic potential of this particular model [30].

## DISCUSSION

An underlying assumption regarding the rationale for using antiangiogenic therapies is that all types of cancer are angiogenesis-dependent and therefore inhibiting angiogenesis would be highly effective as a generic cancer treatment. However, in retrospect this rationale is valid for highly angiogenic types of cancer where VEGF is a key proangiogenic molecule [31] and sunitinib is an established therapy but in BrCa this may not be applicable. Furstenberger *et al*. recently analyzed the expression of a dozen different proangiogenic growth factors in 41 samples of primary BrCa tissue specimens and normal adjacent tissues [32], and discovered that 11/12 factors analyzed showed greater levels of gene expression in the adjacent normal tissue with VEGF being the only exception. These results could expound the shortcomings of antiangiogenic molecules in the various BrCa clinical trials [14, 15] and also highlight the importance of selecting the most appropriate population of patients to treat with a particular agent, since some patients with metastatic BrCa do benefit from antiangiogenic agents over others. Certainly, an important parameter to consider when treating BrCa with antiangiogenic agents is the reported drug induced tumor hypoxia (through upregulation of Hypoxia Inducible Factor, HIF) and an increase in cancer stem cells that promote drug resistance and disease progression [10]. However, a similar effect is also observed for cytotoxic agents [33] suggesting that hypoxia is a key feature in BrCa and combinational treatment regimes including an HIF inhibitor should be evaluated [34]. Thus, several reports justify that antiangiogenic therapy still holds a future in BrCa treatment [13]. It seems plausible that angiogenesis in BrCa may be better addressed by blocking several antiangiogenic signaling pathways, such as STAT, TGF-β or Notch, rather than simply the VEGFR [35].

One such rationally designed and readily conjugatable molecule is presented here (SAP), which proved potent *in vivo* in four different animal models of BrCa. The clinical shortcomings of sunitinib in BrCa suggest that SAP (and similar agents) will most likely be evaluated in a combinational scheme with molecules targeting other critical tumour pathways. Interestingly, *in vitro* data showed that SAP inhibited one such target that is overexpressed in a subset of ER-positive BrCa (RET), and that crosstalk between RET and ER is important in responses to endocrine therapy [38]. Thus SAP could represent a promising molecule active in various ER positive subtypes of BrCa independent of VEGFR inhibition, deserving future investigation as a strategy to prevent endocrine resistance [39].

An important aspect of SAP development was the absence of hematotoxicity in all treated animals, while the observed mild cardiotoxicity was certainly a point of concern irrespective of its severity. We had previously encountered a related cardiotoxic incident with another analogue of sunitinib (SAN1) [18] but this effect was alleviated when SAN1 was conjugated to a peptide moiety. It seems reasonable to assume that the same outcome could be achieved with SAP in future conjugation studies of SAP with a targeting moiety. Such conjugation studies are expected to decrease off-target toxicities and increase tumour biodistribution of the SAP pharmacophore.

A critical aspect of SAP evaluation was its efficacy in syngeneic animal models. A long-standing problem in drug development is the frequent failure of preclinical animal models of disease to accurately predict clinical activity [40, 41]. Although there are important differences between humans and mice, at least this model maintains the homogeneity of BrCa derived from a genetic background similar to the host animal [42], ensuring that the immune response and cancer-host interactions are more faithfully preserved than in the xenograft animal models. Certainly the syngeneic model has its limitations with important differences in the stroma as well as in the innate and adaptive immunity between mice and human, but the fact that SAP inhibited tumour growth is certainly a strong point towards its further development.

In conclusion we have generated a potent sunitinib based analogue characterized by some advantages over the parental molecule including improved solubility, enhanced pharmacokinetic parameters in mice and the ability to be readily conjugated to a targeting peptide or antibody.

## MATERIALS AND METHODS

### Chemicals, synthesis and characterization of SAP

All chemicals were purchased from Sigma-Aldrich, while sunitinib was from Selleckchem, USA. SAP was synthesized as shown in Figure 1 and described in the Supplementary Materials sections (S.I.1). Mass Spectrometry (MS) was employed to define the key spectral features necessary for the quantification of all molecules in blood and tissue as described previously [43, 44]. Total polar surface area (PSA) and clog *P* values were predicted by Chemdraw Ultra (v10, PerkinElmer Informatics). The GST-fusion proteins and the antibodies used for this study are summarized in Supplementary Tables 1 and 2 respectively.

### Computational methods

Detailed docking and molecular dynamic analysis for all the compounds is described in the S.I.2.

### *In vitro* evaluation

The trans-phosphorylation activity of VEGFR-2, PDGFR-β, KIT, FLT3, RET and EGFR was evaluated as described before [45]. Detailed methods and a list of the kinases used are available in the S.I.3. Cellular inhibition of autophosphorylation of VEGFR-2 and PDGFR-β was performed as described in the S.I.4. For cellular studies, cells were used within six months of purchase and were cultured as instructed by the American Type Culture Collection (ATCC-LGC Standards, Germany). All cell lines were obtained from the ATCC while HUVEC were from Life Technologies. E0771 cells were a kind gift of Dr Fernando Rodriguez-Serrano (University of Granada) and were grown as described previously [46]. Cell toxicity was measured by the MTT assay [44, 47].

### Pharmacokinetic analysis

All animal procedures were approved by the Bioethical Committee of BRFAA based on the European Directive 86/609. For pharmacokinetic studies, female NOD/SCID mice (Charles River, Italy) were used (8–10 weeks old, *n* = 6 per group). Dosing solutions of SAP (100 μmol/Kg) were prepared in 20% 2-hydroxypropyl β-Cyclodextrin in sterile water (HP-b-CD). SAP was administered intraperitoneally (IP) or orally and blood samples were collected and prepared as described previously [43].

### *In vivo* efficacy

For the xenograft studies, female NOD/SCID mice were injected in each flank with 3 ×10^6^ HCC1954 (HER2 amplified) or MDA-MB-231 (TNBC) cells, while for the syngeneic animal model 1 × 10^6^ E0771 cells were implanted ectopically in the flanks or orthotopicaly in the fat-pad of the right inguinal mammary gland in female C57BL/6 mice as described before [48]. In every case pharmacological treatment was initiated when tumors reached 100–150 mm^3^ by daily IP administrations of sunitinib or equimolar doses of SAP (100 μmol/Kg, in HP-b-CD). Control mice received HP-b-CD while tumor growth and phenotypic signs of discomfort such as altered behaviour, guarding, mutilation or loss of appetite were constantly monitored during the course of treatment. Experiments were terminated after 18 days, at 1h after the last administered dose by euthanizing the animals under isoflurane anesthesia. Tumors were excised, weighed and prepared for kinase activity, histopathology, immunohistochemistry (IHC) and quantification of compounds of interest by LC-MS/MS.

### Immunohistochemistry

Excised tumors were fixed in neutral buffered formalin, paraffin embedded, sectioned and stained against CD31, Ki-67, pErk1/2 and pS6. Microvessel density was assessed by counting the number of CD31+ vessels in a 40× microscope field in a blinded fashion and presented as the amount of blood vessels/mm^2^. To determine proliferation indices (PI), Ki67 positive or negative cells were counted using ImageJ software (US National Institutes of Health) in 3–4 representative fields of five tumors for each treatment (on average, ~900 nuclei were counted per specimen). Images were acquired by a Leica DFC350-FX camera mounted on a Leica DMLS2 microscope.

### *In vivo* kinase activity

The PathScan intracellular signaling array kit (Cell Signaling, UK) was used as per manufacturer's direction to detect the phosphorylation status in supernatants from tumor extracts (*n* = 10 for each treatment). Images were analyzed with ImageJ software (v1.28) by loading the image as a gray scale picture and the average intensity for each kinase was calculated as described previously [49].

### Toxicity evaluation

C57BL/6 female mice (*n* = 8) were used, treated for one week with daily IP administrations of HP-b-CD or 100 μmol/Kg of SAP or sunitinib. Cardiotoxicity was assessed as described previously [18, 50]. For the hematotoxicity experiments approximately 300 μL of blood were collected from each animal in a vacutainer blood collection tube containing EDTA (BD Biosciences). Blood analysis was performed directly after sampling in a MEK-6318J/K hematology analyzer (Nihon Kohden Corp, Japan) and the parameters measured were red and white blood cell numbers, platelets hemoglobin, hematocrit, mean cell volume (MCV) and mean cell hemoglobin.

### Zebrafish experiments

Zebrafish embryos were maintained and raised as described previously [51]. The experimental protocols described in this study were carried out with zebrafish larvae up to 96 h post fertilization (hpf) and therefore are not subject to the regulations of European animal protection guidelines. The *Tg(kdrl:gfp)*^s483^ transgenic line was used. For the bioaccumulation experiments, synchronized 24 hpf *Tg(kdrl:gfp)*^s483^ embryos were exposed to 50 μM sunitinib or SAP with final concentration 0.1% (v/v) DMSO (vehicle) in embryo medium (0.3 g/L “Instant Ocean” Sea Salts and 0.08 g/L CaSO_4_*2H_2_O). Treatments were performed in three independent experiments with 50 embryos per experimental sample. Embryo and water samples were collected at 1 min and 24 h post treatment. After exposure, embryos were rinsed with fresh embryo medium, snap frozen in liquid nitrogen and stored at −80°C. For monitoring the antiangiogenic activity of SAP and sunitinib *Tg(kdrl:gfp)*^s483^ embryos were treated at 24 hpf with 50 μM of the compounds or vehicle and images were taken at 48 hpf.

### Statistical analyses

Statistical analyses and calculation of all IC_50_ s were performed by SigmaPlot12 software and statistical significance was determined using the Student's two-tailed, two-sample unequal variance distribution *t* test. Comparison of means among three study groups was made by using one-way ANOVA followed by the post hoc Turkey-Kramer multiple comparison test using StatPlus (v6, Analysoft). A two-sided (*P <* .01) was considered statistically significant.

## SUPPLEMENTARY MATERIALS FIGURES AND TABLES


