# Supplementary material for: Targeting of the breast cancer microenvironment with a potent and linkable oxindole based antiangiogenic small molecule
Source: Oncotarget. 2017 Mar 31;8(23):37250–62. doi: 10.18632/oncotarget.16763 (PMC5514907; doi:10.18632/oncotarget.16763)
Supplement: Supplementary file 1 [file oncotarget-08-37250-s001.pdf]

Targeting of the breast cancer microenvironment with a potent and linkable oxindole based antiangiogenic small molecule

Supplementary Materials

SUPPLEMENTARY METHODS 1

Overall Synthesis of SAP

Synthesis of sunitinib analogue SAP commenced from the readily available acid intermediate 1 (Figure 1A of manuscript). Coupling of acid 1 with the Boc-protected piperazine 2 followed by cleavage of the Boc group, resulted in SAP.

Chemicals and experimental procedures

Acid 1 (Figure 1A of manuscript) was purchased from Ontario Chemicals (Guelph, Ontario, Canada) and Deuterated solvents were purchased from Euriso-top (Saint-Aubin Cedex, France). Acetonitrile (LC-MS grade) was purchased from Fisher Scientific (Fisher Scientific, Loughborough, UK) and water (LC-MS grade) was from Carlo Erba (Carlo Erba, Milan, Italy). All commercially available chemicals were used without further purification. All reactions were performed under an argon atmosphere with dry solvents under anhydrous conditions, unless otherwise noted. Methylene chloride (CH_2Cl_2) and dimethylformamide (DMF) were purchased in anhydrous form and used without further purification. Air- and moisture-sensitive liquids were transferred via syringe. Organic solutions were concentrated by rotary evaporation at 40°C. Flash-column chromatography was performed with silica gel 60 (230–400 mesh) as described before (1). Thin layer chromatography (TLC) was performed on pre-coated silica gel 60 F254 plates which were visualized by exposure to ultraviolet light (UV_{254}). ^{1}H NMR spectra were recorded on a 250 MHz Bruker Avance FT-NMR spectrometer. ^{13}C NMR spectra were recorded at 62.9 MHz. Chemical shifts are reported in ppm relative to solvent signal. Multiplicity is indicated as follows: s (singlet); d (doublet); t (triplet); q (quartet); m (multiplet); br (broad); dd (doublet of doublets), ddd (doublet of doublets of doublets). ESI (electron spray ionization) mass spectra were recorded on an Agilent API 4000 QTRAP Series LC/MSD instrument.

Synthesis of Boc-protected analogue 3

Starting from acid 1 and Boc-protected amine 2. A suspension of acid 1 (250 mg, 0.832 mmol) in 10 mL of DMF was treated with EDCI (2 equiv), HOBt (3 equiv) and NE_{t3} (3 equiv) and then stirred at room temperature for 15 min. The resulting clear solution was then treated with amine 2 (2 equiv) and the reaction mixture was stirred at room temperature overnight. After TLC indicated the consumption of acid 1, the reaction mixture was diluted with EtOAc (75 mL) and washed with aq. $NaHCO_3$ (3 × 25 mL). The organic layer was washed with water, dried over Na_2SO_4, filtered and concentrated under reduced pressure to give the crude product. Purification by flash chromatography on silica gel with CH_2C_{l2}:Acetone (from 1:0 to 6:1) afforded compound 3 as an orange solid (60%).

^{1}H NMR ($CDCl_3$): δ 13.43 (s, 1 H), 8.19 (s, 1 H), 7.30 (s, 1 H), 7.16 (dd, 1 H, J = 2.0, 8.5 Hz), 6.89–6.77 (m, 2 H), 6.46 (br s, 1 H), 3.57 (q, 2 H, J = 5.2), 3.47 (s, 4 H), 2.65 (s, 2 H), 2.57 (s, 3 H), 2.53 (s, 4 H), 2.45 (s, 3 H), 1.46 (s, 9 H). MS (ESI$^+$) *m/z* for $C_{27}H_{35}FN_5O_4$: calculated, 512.3; found, 512.3 [M+H$^+$].

Synthesis of SAP

A solution of Boc-protected amine 3 in CH_2Cl_2 was treated with trifluoroacetic acid (10 equiv) and the reaction mixture was stirred at room temperature (3–5 h). After TLC indicated the consumption of starting material, the reaction mixture was diluted with CH_2Cl_2 and washed with aq. $NaHCO_3$. The organic layer was washed with water, dried over Na_2SO_4, filtered and concentrated under reduced pressure to give the crude product. Purification by flash chromatography on silica gel with CH_2Cl_2:CH_3OH (from15:1 to 5:1) afforded the pure compound as an orange solid (90% yield).

^{1}H NMR (DMSO-d_6): δ 13.75 (s, 1 H), 10.92 (s, 1 H), 7.81–7.69 7.77 (dd, 1 H, J = 2.3, 9.8 Hz), 7.73 (s, 1 H), 7.71 (t 1 H, J = 5.5 Hz), 6.99–6.81 (m, 2), 3.54 (q, 2 H, J = 5.7 Hz), 3.33 (s, 8 H), 3.16 (m, 2 H), 2.47 (s, 3 H), 2.45 (s, 3 H). MS (ESI$^+$) *m/z* for $C_{22}H_{27}FN_5O_2$: calculated, 412.2; found, 412.3 [M+H$^+$].

SUPPLEMENTARY METHODS 2

Computational analysis

The crystal structures of VEGFR-2 juxtamembrane and kinase domains in complex with sunitinib (PDB ID 4AGD) (2) and the kinase domain of KIT in complex with sunitinib (PDB ID 3G0E) (3) were retrieved from

the RCSB PDB (4). For the three protein residues of KIT with alternative locations, only the highest occupancy conformation was retained. The homology model of PDGFR-β kinase domain (residues 600–962) was prepared using MODELLER (v9.10) (5). The highly homologous VEGFR-2 kinase domain (~50%) was employed as template using the alignment described previously (6). One hundred models were generated and the PDGFR-β model with the lowest DOPE score (7) was selected for docking calculations. Non-protein atoms were removed and all structures were superimposed using the MultiSeq module (8) of VMD (v1.9) (9). The initial conformations of the ligands were generated from the crystallographic structure of sunitinib bound to the ATP site of VEGFR-2 using the OpenEye program VIDA (v.4.1, OpenEye Scientific Software) by substituting the triethylamine moiety of the ligand with the corresponding linker. The piperazine ring of SAP ligand was protonated at the terminal amino group, all non-polar hydrogen atoms of the protein and the ligands were removed, and Gasteiger charges were applied using AutoDockTools (v 1.5) (10). The search space was defined by a grid box centered on sunitinib and comprised 81×81×81 grid points of 0.375 Å spacing. For each complex, 100 docking rounds were calculated using AutoDock (v4.2) and the Lamarckian genetic algorithm with the default parameters (11). The maximum number of energy evaluations was set to 10 million and the results were clustered using a tolerance of 2.0 Å. Calculations were carried out using Intel Xeon workstations equipped with NVIDIA GTX-780 GPUs and operated by Linux 2.6.32 kernels.

SUPPLEMENTARY METHODS 3

Biochemical tyrosine kinase assay

The trans-phosphorylation activity of RTKs VEGFR-2, PDGFR-β, KIT, RET, FLT-3 and EGFR was investigated as described before (12). A detailed list of the GST-Fusion proteins used is given in Supplementary Table 1. Briefly, reactions are performed in 96-well microtiter plates precoated (20 μg/well in PBS; incubated overnight at 4 °C) with the peptide substrate poly-Glu,Tyr (4:1). Excess protein binding sites are blocked with the addition of 5% (w/v) BSA in PBS. Purified GST-fusion proteins are then added to the microtiter wells in 2 × concentration kinase dilution buffer consisting of 100 mM HEPES, 50 mM NaCl, 40 μM $NaVO_4$, and 0.02% (w/v) BSA. The final enzyme concentration for each GST-kinase is 50 ng/mL. Twenty-five μL of diluted sunitinib or each SAN are subsequently added to each reaction well to produce a range of inhibitor concentrations appropriate for each enzyme. The kinase reaction is initiated by the addition of different concentrations of ATP in a solution of $MnCl_2$ so that the final ATP concentrations spanned the Km for the enzyme, and the final concentration of $MnCl_2$ is 10 mM. The plates are incubated for 60 minutes at 37°C before stopping the reaction with the addition of EDTA. The plates are then washed three times with TBST. Rabbit polyclonal antiphosphotyrosine antisera conjugated to HRP is added to the wells at a 1:1000 dilution in TBST containing 0.5% (w/v) BSA, 0.025% (w/v) nonfat dry milk, and 100 μM $NaVO_4$ and incubated for one hour at 37°C. The plates are then washed three times with TBST and the amount of phosphotyrosine in each well is quantified after the addition of 2,2′-azino-di-[3-ethylbenzthiazoline sulfonate] substrate (A1888, Sigma-Aldrich, Munich, Germany) and measured at 405 nm. Each experiment was performed in duplicate in three individual experiments.

SUPPLEMENTARY METHODS 4

Cellular autophosphorylation assay

HUVE cells (for VEGFR-2) and NIH/3T3 (for PDGFR-β) were used in studies of inhibition of autophosphorylation. Serum starved cells were treated with DMSO or drugs (concentrations ranging from 0.001 to 1 μM) for 2 h and then stimulated with 50 ng/mL VEGF165 or PDGF-BB (H9166 and SRP3229 both from Sigma-Aldrich, Munich, Germany) for 10 min at 37°C before cell harvesting in radioimmunoprecipitation assay (RIPA) buffer containing protease and phosphatase inhibitors. Protein isolated from HUVEC or NIH/3T3 cell lysate (approximately 50 μg of protein/cell line), along with NuPAGE Sample Reducing agent and NuPAGE LDS Sample Buffer (both from Invitrogen, UK) were separated by SDS page and transferred to polivinylidene difluoride (PVDF) membranes. Phosphorylated and total VEGFR-2 and PDGFR-β were detected using specific antibodies at 1:1000 dilution in TBST (a detailed list of all antibodies is given in Supplementary Table 2). Secondary, HRP labelled antibody was used at 1:2000 dilution, followed by Enhanced Chemilluiminescence (GE Healthcare, UK) visualization. For *in vivo* experiments, 100 μg of tumor tissue were homogenized in RIPA buffer and treated as above. The intensity of each band was calculated using ImageJ Software analysis (v1.47, NIH, USA).

SUPPLEMENTARY METHODS 5

Would healing/migration assay

MDA-MB-231 cells were seeded at a density of 10^5 in 24 well plates and cultured for 24 h. Monolayers were wounded using the tip of a pipette, washed by PBS, and further incubated in DMEM medium with 10% FBS in the presence or absence of sunitinib or SAP at different concentrations for 24 h. Images were acquired via a phase-contrast microscope and the wound width was measured at various time points.

REFERENCES

1. Still WC, Kahn M, Mitra A. Rapid chromatographic technique for preparative separations with moderate resolution. The Journal of Organic Chemistry. 1978; 43:2923–5.
2. McTigue M, Murray BW, Chen JH, Deng YL, Solowiej J, Kania RS. Molecular conformations, interactions, and properties associated with drug efficiency and clinical performance among VEGFR TK inhibitors. Proceedings of the National Academy of Sciences. 2012; 109:18281–9.
3. Gajiwala KS, Wu JC, Christensen J, Deshmukh GD, Diehl W, DiNitto JP, English JM, Greig MJ, He YA, Jacques SL, Lunney EA, McTigue M, Molina D, et al. KIT kinase mutants show unique mechanisms of drug resistance to imatinib and sunitinib in gastrointestinal stromal tumor patients. Proceedings of the National Academy of Sciences. 2009; 106:1542–7.
4. Berman HM, Westbrook J, Feng Z, Gilliland G, Bhat TN, Weissig H, Shindyalov IN, Bourne PE. The Protein Data Bank. Nucleic Acids Res. 2000; 28:235–42.
5. Sali A, Blundell TL. Comparative Protein Modelling by Satisfaction of Spatial Restraints. Journal of Molecular Biology. 1993; 234:779–815.
6. Argyros O, Karampelas T, Asvos X, Varela A, Sayyad N, Papakyriakou A, Davos CH, Tzakos AG, Fokas D, Tamvakopoulos C. Peptide-drug conjugate GnRH-sunitinib targets angiogenesis selectively at the site of action to inhibit tumor growth. Cancer Res. 2015; 76:1181–92.
7. Shen MY, Sali A. Statistical potential for assessment and prediction of protein structures. Protein Science. 2006; 15:2507–24.
8. Roberts E, Eargle J, Wright D, Luthey-Schulten Z. MultiSeq: unifying sequence and structure data for evolutionary analysis. BMC Bioinformatics. 2006; 7:382.
9. Humphrey W, Dalke A, Schulten K. VMD: Visual molecular dynamics. Journal of Molecular Graphics. 1996; 14:33–8.
10. Sanner MF. Python: a programming language for software integration and development. J Mol Graph Model. 1999; 17:57–61.
11. Huey R, Morris GM, Olson AJ, Goodsell DS. A semiempirical free energy force field with charge-based desolvation. Journal of Computational Chemistry. 2007; 28:1145–52.
12. Sun L, Liang C, Shirazian S, Zhou Y, Miller T, Cui J, Fukuda JY, Chu JY, Nematalla A, Wang X, Chen H, Sistla A, Luu TC, et al. Discovery of 5-[5-Fluoro-2-oxo-1,2- dihydroindol-(3Z)-ylidenemethyl] -2,4- dimethyl-1H-pyrrole-3-carboxylic Acid (2-Diethylaminoethyl) amide, a Novel Tyrosine Kinase Inhibitor Targeting Vascular Endothelial and Platelet-Derived Growth Factor Receptor Tyrosine Kinase. Journal of Medicinal Chemistry. 2003; 46:1116–9.

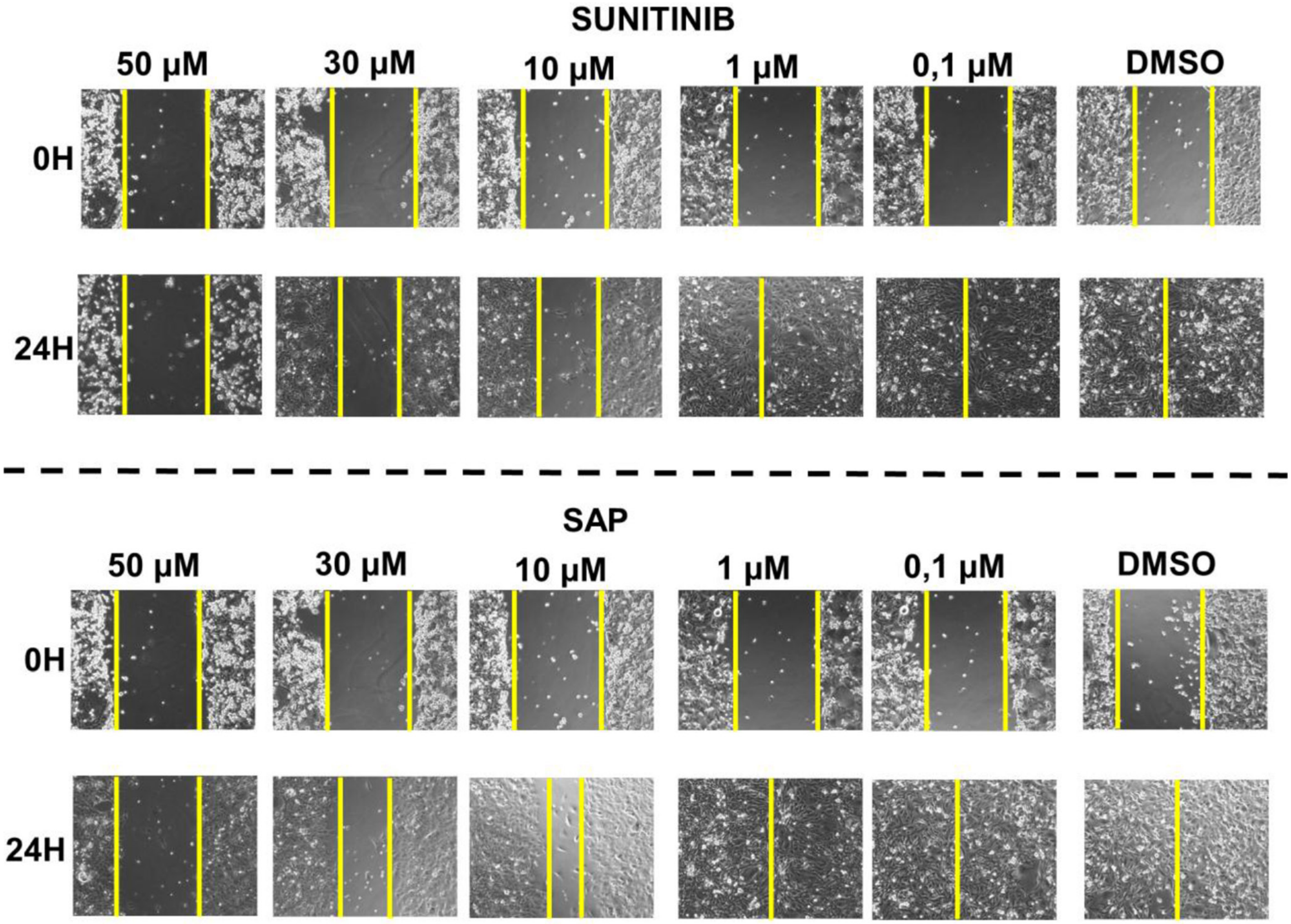

Supplementary Figure 1: Wound healing assay. Sunitinib and SAP inhibited MDA-MB-231 migration in wound healing assay. Cells were wounded by the pipette tip and then treated with various concentrations of compounds for 24 hours. Control cells (labeled DMSO) were treated with 0.1% DMSO.

Supplementary Table 1: List of GST- fusion proteins used in this study

	VEGFR-2	PDGFR-β	KIT	FLT-3	RET	EGFR
Supplier	Sigma Aldrich	Sigma Aldrich	Sigma Aldrich	Sigma Aldrich	Sigma Aldrich	Sigma Aldrich
Cat #	K2643	G8671	C0624	F6432	R7782	SRP5023
Kinase Domain	789-end	557-end	544-end	571–993	658-end	695-end

Supplementary Table 2: List of antibodies used in this study

Target	Phosphosite	Company	Cat#	Application
VEGFR-2	Tyr1175	Cell Signaling	2478	WB
PDGFR-β	Tyr751	Cell Signaling	4549	WB
VEGFR-2	N/A	Cell Signaling	D5B1	WB
PDGFR-β	N/A	Cell Signaling	3169	WB
Erk1/2	Thr202/Tyr204	Cell Signaling	4370	WB
CD31	N/A	AbCam	ab28364	IHC
Ki-67	N/A	AbCam	ab15580	IHC
pS6	Ser235/Ser236	Cell Signaling	2211	IHC
Antiphosphotyrosine conjugated to HRP	N/A	AbCam	ab9329	Kinase Assay
